# Cardiac safety of off-label COVID-19 drug therapy: a review and proposed monitoring protocol

**DOI:** 10.1177/2048872620922784

**Published:** 2020-04-01

**Authors:** Niyada Naksuk, Sorin Lazar, Thoetchai (Bee) Peeraphatdit

**Affiliations:** Division of Cardiology, University of Illinois at Chicago, USA; Center for Liver Diseases, The University of Chicago Medicine, USA

**Keywords:** COVID-19, treatment, drugs, adverse effects, cardiac, arrhythmias

## Abstract

More than 2,000,000 individuals worldwide have had coronavirus 2019 disease infection (COVID-19), yet there is no effective medical therapy. Multiple off-label and investigational drugs, such as chloroquine and hydroxychloroquine, have gained broad interest due to positive pre-clinical data and are currently used for treatment of COVID-19. However, some of these medications have potential cardiac adverse effects. This is important because up to one-third of patients with COVID-19 have cardiac injury, which can further increase the risk of cardiomyopathy and arrhythmias. Adverse effects of chloroquine and hydroxychloroquine on cardiac function and conduction are broad and can be fatal. Both drugs have an anti-arrhythmic property and are proarrhythmic. The American Heart Association has listed chloroquine and hydroxychloroquine as agents which can cause direct myocardial toxicity. Similarly, other investigational drugs such as favipiravir and lopinavir/ritonavir can prolong QT interval and cause Torsade de Pointes. Many antibiotics commonly used for the treatment of patients with COVID-19, for instance azithromycin, can also prolong QT interval. This review summarizes evidenced-based data regarding potential cardiac adverse effects due to off-label and investigational drugs including chloroquine and hydroxychloroquine, antiviral therapy, monoclonal antibodies, as well as common antibiotics used for the treatment of COVID-19. The article focuses on practical points and offers a point-of-care protocol for providers who are taking care of patients with COVID-19 in an inpatient and outpatient setting. The proposed protocol is taking into consideration that resources during the pandemic are limited.

## Introduction

We are in the middle of the coronavirus disease 2019 (COVID-19) pandemic and it is predicted that nearly 500 million individuals worldwide will be infected.^1^ As of April 2020, the mortality rate in each country ranges from 1% to 13%.^2^ While large scale studies are being conducted in multiple countries, their preliminary results on effective therapies are at least a few months ahead. Awaiting the results from clinical trials, providers across the globe are using off-label and investigational drugs with unknown safety profiles.

## Safety concerns in patients with COVID-19

Emerging data have shown that cardiovascular comorbidities are very common in patients with COVID-19 and such patients are at increased risk of death.^3^ Furthermore, 19–33% of hospitalized patients with COVID-19 have concurrent cardiac injury.^4–6^ The mechanism may include severe systemic inflammatory responses, direct injury from the severe acute respiratory syndrome coronavirus 2 (SARS-CoV-2), hypoxia or microthrombi leading to microvascular damage.^7^ However, adverse effects from pharmacotherapy cannot be entirely excluded. In addition, concomitant cardiac injury from SARS-CoV-2 infection may increase the risk of adverse events from generally safe drugs.^8^ For instance, patients with cardiomyopathy and/or congestive heart failure have reduced repolarization reserve and are at increased risk of drug-related proarrhythmic risk.^8,9^

Other specific concerns during the COVID-19 pandemic may include lack of adequate cardiac testing giving a shortage of healthcare providers and ancillary staff, as well as the intention to minimize the risk of exposure. Finally, when using off-label medications to treat novel disease such as COVID-19, drug–drug interaction can be underestimated.

## Chloroquine and hydroxychloroquine

Among those investigational drugs, antimalarial and anti-rheumatic drugs, namely chloroquine and hydroxychloroquine, respectively, have gained broad interest. In an in vitro study, chloroquine 500 mg twice daily and hydroxychloroquine 400–600 mg twice a day loading followed by 400–600 mg blocked SARS-CoV-2 cell entry in vitro.^10^ In addition, an early study suggested clinical benefit in patients with COVID-19, showing reduction in pneumonia severity, length of hospitalization, and viral shedding.^11^

Despite generally safe profiles of chloroquine and hydroxychloroquine when used at low dose, both drugs can have significant cardiovascular adverse effects. Reports from long-term users with a smaller daily dosage found a broad prevalence of cardiac toxicity in the form of mild to severe conduction disorders and irreversible cardiomyopathy. The cumulative dose range (15–5040 g) and duration of treatment (7 months –35 years) vary greatly.^12^ Severe and irreversible cardiac damage has been reported. Hydroxychloroquine may have less toxicity, but is not without risk.

Chloroquine and hydroxychloroquine are proarrhythmic and can cause significant QT prolongation, as well as increasing the risk of Torsade de Pointes (TdP) even at therapeutic doses.^13^ They are generally contraindicated in patients with congenital long QT syndrome or those who have a prior history of TdP. Other electrocardiographic changes may include T-wave inversion or depression. In healthy animal models, both agents, especially chloroquine, decreased excitability and conductivity of atrial and ventricular myocardium, although the magnitude is much less than quinine or quinidine, a related class I anti-arrhythmic drug.^14^ Nonetheless, chloroquine and hydroxychloroquine have been shown to be effective in the acute suppression of wide ranges of atrial and ventricular arrhythmias.^13^ A study of 28 patients taking 250 mg daily of chloroquine found QT (Qtc) interval lengthened from 363–388 milliseconds to 372–392 milliseconds.^15^ The dose recommended for the treatment of COVID-19 is 500 mg twice a day, therefore the risk of QT prolongation is expected to be higher. Furthermore, case reports of chloroquine or hydroxychloroquine toxicity observed widened QRS complex due to their excessive I_Na_ blockage property. A study of 72 subjects with and without structural heart disease given acute chloroquine and hydroxychloroquine therapy for various types of atrial and ventricular arrhythmias observed one sudden death.^13^ The dosage used in the study was slightly higher than that proposed in the treatment of COVID-19. Conclusively, the American Heart Association has listed chloroquine and hydroxychloroquine as agents which can cause direct myocardial toxicity and exacerbate underlying myocardial dysfunction.^16^

## Antiviral drugs

**Figure 1.**
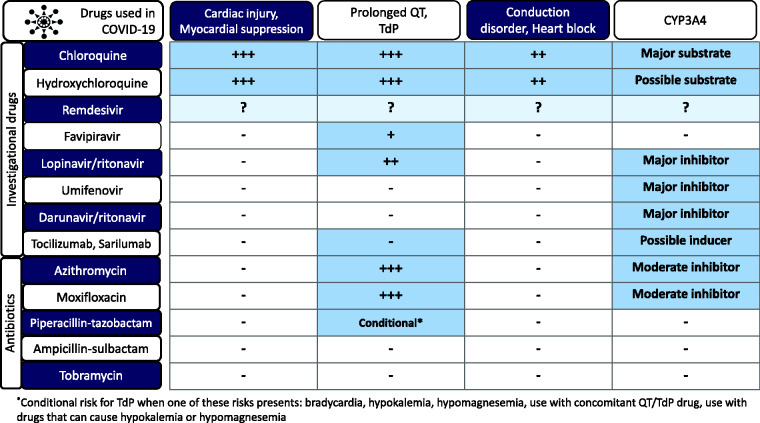
Potential cardiac adverse effects and drug interaction in investigational drugs and common antibiotics used in the treatment of COVID-19 COVID-19: coronavirus disease 2019; TdP: Torsade de Pointes.

Current pharmacotherapeutic regimens for COVID-19 may include investigational and off-label drugs, such as a novel antiviral drug namely remdesivir, with unknown clinical safety (Figure 1). Lopinavir/ritonavir combination remains a treatment regimen for COVID-19 in many countries although no clinical benefit was observed in a small study.^17^ Importantly, lopinavir/ritonavir can cause QT prolongation and are CYP3A4 inhibitors,^17,18^ and cannot be used with major CYP3A4 substrates such as chloroquine. Prolonged QT interval due to favipiravir, which is now being used for the treatment of COVID-19 in some regions, has been reported.^19^ Umifenovir and darunavir for the treatment of influenza have a safe cardiac profile. However, darunavir/ritonavir is a major CYP3A4 inhibitor.^20^

## Monoclonal antibodies

Tocilizumab and sarilumab are interleukin-6 (IL-6) inhibitors approved for the treatment of rheumatoid arthritis. Both drugs are not associated with heart failure exacerbation or proarrhythmia. Interestingly, case series observed shortening of the QT interval (to <440 milliseconds) in patients with rheumatoid arthritis who were treated with tocilizumab for more than 3 months.^21,22^ While the mechanism was unclear, it was thought to be from a decrease in inflammation in cardiomyocytes.^22^ Furthermore, tocilizumab was used safely among patients with autoimmune disease and cardiomyopathy.^23,24^

## Antibiotics

The combination of chloroquine or hydroxychloroquine or antiviral drugs with other antibiotics is a common practice. Careful QT interval monitoring should be performed when concomitant macrolide drugs (such as azithromycin, moxifloxacin) are administered.^25,26^ Furthermore, piperacillin -tazobactam carries some risk of TdP, especially when one of additional risks or TdP presents (i.e. bradycardia, hypokalemia, hypomagnesemia, use with concomitant QT/TdP drugs, use with drugs that can cause hypokalemia or hypomagnesemia).

## Pharmacokinetic consideration

Chloroquine and hydroxychloroquine are metabolized via CYP2C8 and CYP3A4,^27^ therefore caution should be focused on potential drug interactions (Figure 1) and patients with hepatic disease. Chloroquine is substantially excreted by the kidney and the risk of toxicity may be greater in patients with impaired renal function, although specific dosage adjustment may not be required. Chloroquine significantly reduces the bioavailability of ampicillin. An interval of at least 2 hours between the intake of ampicillin and chloroquine is recommended.^28^ Hydroxychloroquine has a remarkably long half-life of 40–50 days.^29^ Multiple drugs being used for the treatment of COVID-19 are CYP3A4 inhibitors which can significantly increase serum chloroquine and hydroxychloroquine (Figure 1). In particular, the combination of chloroquine and lopinavir/ritonavir, or umifenovir/ritonavir is contraindicated.^20^

Many cardiovascular drugs such as ticagrelor, rivaroxaban and apixaban are major substrates of CYP3A4. Co-administration with CYP3A inhibitors may require a dose adjustment or additional monitoring. Furthermore, warfarin concentrations are decreased when co-administered with ritonavir. It is recommended that the international normalized ratio (INR) be monitored more frequently when warfarin is combined.

Cytochrome P450s are generally downregulated by infection and inflammation stimuli including cytokines such as IL-6. Inhibition of IL-6 signaling treated with tocilizumab or sarilumab may restore CYP450 activities to higher levels leading to increased metabolism of drugs that are CYP450 substrates. The effects may last up to one week following a single dose of IL-6 inhibitor and are clinically relevant for CYP450 substrates with a narrow therapeutic index, in which the dose is individually adjusted such as warfarin.

## Proposed point-of-care protocol for inpatient cardiac work-up and monitoring

**Figure 2.**
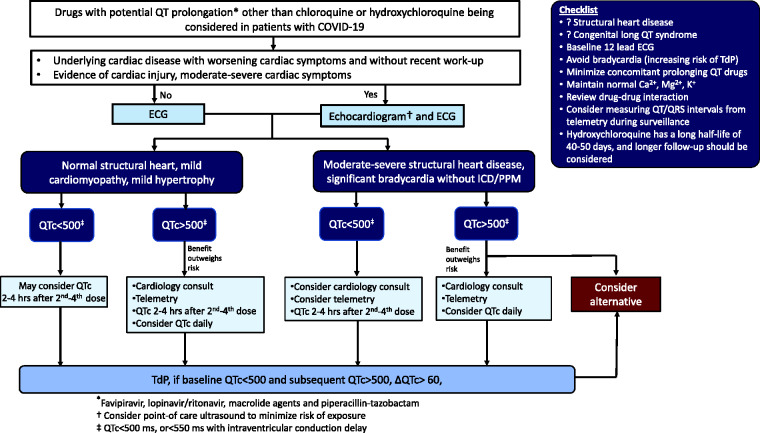
Proposed protocol for initiation and monitoring of drugs with potential QT prolongation used in hospitalized patients with COVID-19 COVID-19: coronavirus disease 2019; ECG: electrocardiogram; ICD: implantable cardioverter defibrillator; PMM: pacemaker implantation; TdP: Torsade de Pointes.

**Figure 3.**
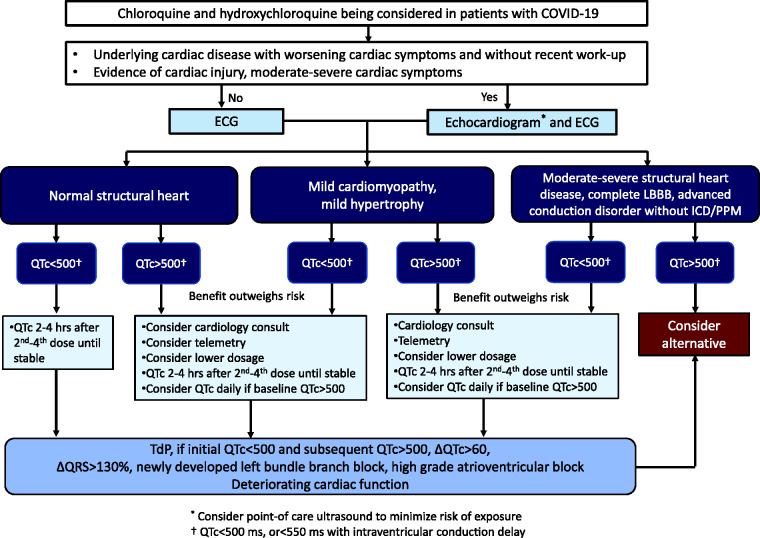
Proposed protocol for initiation and monitoring chloroquine and hydroxychloroquine used in hospitalized patients with COVID-19 COVID-19: coronavirus disease 2019; ECG: electrocardiogram; ICD: implantable cardioverter defibrillator; PMM: pacemaker implantation; TdP: Torsade de Pointes.

Our proposed work-up and monitoring protocol for drugs commonly used for the treatment of COVID-19 is shown in Figure 2 and for chloroquine and hydroxychloroquine in Figure 3, respectively. The algorithms are in line with the recently published guidance from the Mayo Clinic^30^ and from the American College of Cardiology magazine which focus solely on hydroxychloroquine and azithromycin.^8^ Our protocol has extended those statements by taking underlying cardiomyopathy into consideration, as well as covering other drugs currently used in the treatment of the pandemic. Generally, drugs with potential QT prolongation should be avoided in patients with QTc greater than 500 milliseconds (or 550 milliseconds with intraventricular conduction delay) especially in the setting of moderate to severe structural heart disease and patients with high-grade atrioventricular block without a pacemaker and/or implantable cardioverter defibrillator in place.^31,32^ In patients with baseline QTc greater than 500 milliseconds and normal or mild cardiomyopathy, benefit may outweigh the risk and shared decision-making should be discussed. The checklist shown in Figure 2 should be applied for all patients. This includes minimizing concomitant prolonging QT drugs and concomitant CYP3A4 inhibitors and substrates, and maintaining normal serum electrolyte levels. Bradycardia can precipitate TdP and thus conditions or medications with the potential to slow the heart rate should also be avoided. These following criteria should prompt dose reduction or drug discontinuation: subsequent QTc interval greater than 500 milliseconds (or 550 milliseconds with intraventricular conduction delay) in those with baseline QTc less than 500 milliseconds; ΔQTc greater than 60 milliseconds.^30,32^ A preliminary report showed that the maximal change in the QT interval among patients with COVID-19 treated with combined hydroxychloroquine/azithromycin occurred between days 3 and 4.^33^ Thus, in a setting with limited resources, QTc interval measurement may be performed on days 3–4.

Because chloroquine and hydroxychloroquine can cause a broader spectrum of cardiac adverse effects (including suppression of myocardial function and heart block), the proposed protocol in Figure 3 and criteria for dose adjustment are slightly different from other drugs. In particular, the QTc interval should be measured more frequently.^8^ Subsequent surveillance can be flexible and based on: (a) underlying cardiac disease and function; (b) baseline QTc interval; (c) underlying conduction disorder; and (d) other risk factors for TdP. In addition to the above parameters, ΔQRS greater than 130%; high grade atrioventricular block, or newly developed left bundle branch block should prompt dose reduction or drug discontinuation.

While a 12-lead electrocardiogram (ECG) is considered the gold standard for QT measurement, to limit the chance of viral transmission to healthcare providers, using cardiac telemetry or a wireless device for calculating QT interval may be applied after initial comparison is acceptable.^30,34^

## Proposed monitoring protocol for use of chloroquine and hydroxychloroquine in an outpatient setting

**Figure 4.**
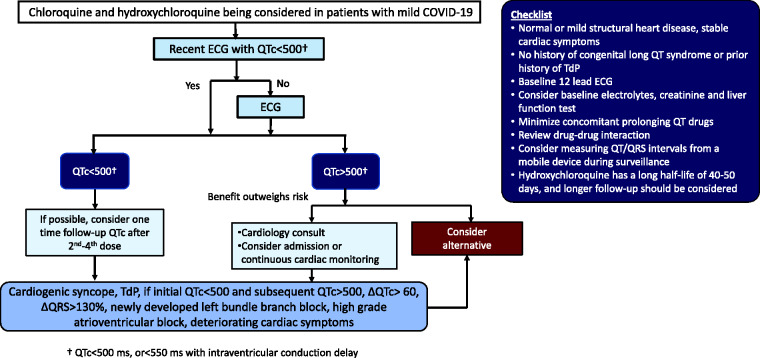
Proposed monitoring protocol for use of chloroquine and hydroxychloroquine in the outpatient setting COVID-19: coronavirus disease 2019; ECG: electrocardiogram; TdP: Torsade de Pointes.

Chloroquine and hydroxychloroquine may be used for the early treatment of patients with mild COVID-19 who are stable and do not require hospitalization. Providers should ensure patients’ history without significant underlying cardiomyopathy or cardiac symptoms. If a recent 12-lead ECG is not available, a new baseline ECG should be obtained. In addition, liver function test, serum creatinine and serum electrolytes may be considered in patients at risk. Figure 4 presents the proposed monitoring protocol with an initial checklist. In patients with baseline QTc less than 500 milliseconds, consider one-time QTc interval measurement after the second to fourth dose. In order to limit the risk of exposure, a mobile or wireless device should be considered for this purpose.

## Conclusion

Multiple pharmacotherapeutic agents including chloroquine, hydroxychloroquine, antiviral drugs and monoclonal antibodies are being investigated for the treatment of patients with SARS-CoV-2 infection. Based on available data, chloroquine and hydroxychloroquine can cause irreversible cardiomyopathy and worsen pre-existing myocardial dysfunction. Other known cardiac side effects include high-grade atrioventricular block, bundle branch block, QT prolongation and TdP. Furthermore, favipiravir, lopinavir/ritonavir, macrolide agents and piperacillin–tazobactam can cause prolonged QT and TdP. Particular concerns in patients with COVID-19 include underlying structural heart disease, cardiac injury, renal and hepatic dysfunction, limited resource for cardiac monitoring and drug–drug interaction. Clear administration protocols should be in place in every hospital and clinic using these drugs for the treatment of COVID-19.

## Take-home points

- Multiple investigational and off-label drugs with uncertain cardiac safety profiles are currently used for the treatment of COVID-19, which has caused infection in over 1,000,000 individuals worldwide. This is an important topic because patients with COVID-19 often have underlying cardiovascular disease and up to 33% of hospitalized patients have cardiac injury.
- The medication list includes anti-malaria and anti-rheumatic drugs (chloroquine and hydroxychloroquine, respectively), antiviral drugs, as well as monoclonal antibodies. In addition, antimicrobial agents such as macrolides are frequently prescribed.
- Of the off-label drug regimens for the treatment of COVID-19, chloroquine, hydroxychloroquine, favipiravir, lopinavir/ritonavir and macrolides can cause significant QT prolongation and TdP.
- Furthermore, chloroquine and hydroxychloroquine directly suppress myocardial function and can cause heart block. Both agents are listed as a direct cause of cardiac toxicity by the American Heart Association.

## Conflict of interest

The author(s) declared no potential conflicts of interest with respect to the research, authorship, and/or publication of this article.
